# The Human Interference Scoring System (HISS): A New Tool for Quantifying Food Quality Based on Its Level of Processing

**DOI:** 10.3390/nu16040536

**Published:** 2024-02-14

**Authors:** Olivia Malamatenios, Jessica L. Campbell, Grant Schofield, Caryn Zinn

**Affiliations:** Human Potential Centre, Faculty of Health and Environmental Sciences, Auckland University of Technology, Auckland 1142, New Zealand

**Keywords:** food-classification systems, food quality, ultra-processed foods, food processing, minimally processed foods, reliability

## Abstract

The Human Interference Scoring System (HISS) is a novel food-based diet-quality-classification system based on the existing NOVA method. HISS involves food and fluid allocation into categories from digital imagery based on food processing levels, followed by meal plan analysis using food-servings quantification. The primary purpose of this work was to evaluate the reliability of HISS. Trained nutrition professionals analyzed digital photographs from five hypothetical 24 h food recalls and categorized foods into one of four HISS categories. A secondary purpose was to assess the nutrient composition of the food recalls and other selected foods from the HISS categories. Participants effectively categorized foods into HISS categories, with only minor discrepancies noted. High inter-rater reliability was observed in the outer HISS categories: unprocessed and ultra-processed foods. Ultra-processed items consistently displayed elevated energy, carbohydrates, and sugar compared to unprocessed foods, while unprocessed foods exhibited notably higher dietary fiber. This study introduces the HISS as a potentially useful tool for quantifying a food-quality-based system using digital-photography-based assessments. Its high inter-rater reliability and ability to capture relationships between food processing levels and nutrient composition make it a promising method for assessing dietary habits and food quality.

## 1. Introduction

A significant portion of the global disease burden is now of chronic disease, with dietary factors known to contribute to several leading causes of death [1,2]. However, ongoing debates in nutrition science about nutrient-based dietary recommendations complicate measuring diet healthfulness. Traditional diet-quality indices are based on quantifying nutrients, food groups, or nutrient density, according to nationally and internationally developed individual nutrient thresholds. This is one way to assess diet quality, yet it does not differentiate between whole or processed foods. For example, a diet rich in whole foods might be rated similarly to a highly processed, fortified food diet when assessed based on individual nutrients. Further, this approach does not consider the food matrix. Retrieving dietary information from individuals in nutrition research is reliant on food recalls which are onerous, require some level of nutrition literacy, and which suffer from inherent bias [3,4]. Alternative classification systems based on the level of food processing such as NOVA have been proposed [5,6]. While appearing promising, these still suffer from difficulties in interpretation and have high inter-rater variability [7]. As such, there remains a need to develop a system that is based on sound and agreed-upon nutritional principles which move beyond single nutrient models of human health. Any such system should allow for easier translation into a public health setting and, importantly, provide a way to interact with the public that is more intuitive.

There is growing consumer interest in the nutritional value of diets, but this is accompanied by public confusion and professional debates about what constitutes a healthy diet [8,9,10,11]. It is also important to recognize that healthy diets can vary greatly in macronutrient ratios across different dietary patterns. Evidence-backed consensus [12,13,14,15,16,17] emphasizes that diets rich in minimally processed foods are optimal for human and planetary health [1,18,19]. Despite this consensus, global diets are increasingly shifting towards including more processed foods [20,21,22,23,24]. While some food processing is beneficial for safety, preservation, and allowing supply during off-seasons [25,26], many modern food systems prioritize profit over optimal nutrition, leading to nutrient-poor, energy-dense processed foods that are highly palatable and cheap [27].

A notable shift is the rise in ‘ultra-processed’ foods (UPFs), which tend to be energy-dense, low in essential nutrients, and high in refined starches, sugars, fats, additives, and salt [12]. As defined by Monteiro [28] (p. 1476)**,** they are ‘industrial formulations made mostly or entirely with substances extracted from foods, often chemically modified, and from additives, with little if any whole food added.’ While not recommended for prolonged consumption, the hyper-palatability, texture, and availability of many UPFs, alongside aggressive digital marketing strategies are increasing their regular consumption [16,29,30]. This trend is contributing to rising levels of non-communicable diseases worldwide [27]. Studies indicate that high UPF intake is correlated with increased health risks [12,13,16,17,31]. This correlation persists despite criticism [32,33] and is not fully explained using traditional dietary models focusing on high fat and sugar content [29,34]. Moreover, particularly due to their often-soft texture, UPFs potentially disrupt normal signaling related to satiety and appetite, therefore, predisposing individuals to over-consumption [14,29,30].

Various food-classification systems with different categories, definitions, and applicability aim to categorize foods using their processing levels. NOVA [5,6], the most widely used system, has been adopted as part of Brazil’s visionary dietary guidelines. These guidelines emphasize a holistic approach considering environmental impact, cultural factors, and the context of food consumption, beyond individual nutrients [35]. However, despite its popularity, the NOVA system suffers from interpretational ambiguity and categorization inconsistencies [7,36]. Moreover, NOVA group 2, which encompasses culinary ingredients like sugar and oils, prevents the system from showing a clear progression in processing levels from the least to most processed, as expected across its four categories. These issues, coupled with self-reporting biases, which tend to increase with higher levels of body fat [3,4], underscore the need for a more robust alternative.

Here, we present the Human Interference Scoring System (HISS). HISS builds on NOVA in three ways: (i) it incorporates a progressing food processing category system; (ii) it utilizes digital imagery for food recalls; and (iii) it introduces the application of serving sizes for meal plan quantification purposes. This study tests HISS’s reliability and broader utility, exploring its efficacy in categorizing food processing levels and quality using digital imagery. We aimed to address the following questions:

1. Can nutrition professionals understand how to use the HISS to categorize food from digital food photography?
2. Can the HISS be used to identify the degree of processing, eating patterns, and, therefore, accurately reflect the measure of food quality when analyzing digital images?
3. What is the usability of the HISS and what improvements can be made to the food-classification system?
4. What is the relationship between HISS categories and the nutrient composition of foods within them?

## 2. Materials and Methods

### 2.1. Design and Development of a Food Processing Classification System

The Human Interference Scoring System (HISS) was designed and developed by a team of researchers working in the Human Potential Centre at Auckland University of Technology (AUT). HISS allows users to assess the degree of processing in food items and meals. It also helps quantify the amount of food within each HISS category, focusing on food quality assessment. After an in-depth review of the literature assessing commonly used food-classification systems applied in research settings, the HISS classification system was developed based on a modification to the present NOVA system. Like NOVA, HISS was developed with four categories. Each category clearly describes the degree of processing and includes the types of food and drink it encompasses. Category 1 and 4 in HISS and NOVA have similar specifications, in that category 1 is unprocessed and minimally processed food, and category 4 comprises ultra-processed foods. HISS also does not include a category exclusively for certain ingredients (as does NOVA in category 2 for culinary ingredients). Common examples of food and drink items meeting these criteria are provided (Table 1).

HISS was designed with four categories to minimize the crossover common in systems with more categories. This design enhances the distinction between the levels of food processing and allows for the inclusion of home-cooked items. HISS defines food processing as any method or process that alters food from its raw and natural state, transforming the original food matrix into either safer, more digestible, or more palatable food products. HISS also differs from NOVA in the removal of a designated culinary ingredients category.

Although some food processing classifications have been used in a quantitative manner by assessing the percentage of nutrients coming from each group [37], HISS was developed to more explicitly allow for quantitative assessment. Serving sizes for each food or drink were incorporated into the framework. This enables the calculation of the total number of servings, both overall and per category, after assigning each food to the correct HISS category. Further, this enables HISS to provide a comprehensive representation of dietary patterns, food quality, and the overall healthiness of meals/diets. In the majority of cases, national food guidelines’ serving sizes (in this case the Ministry of Health (MOH)) were utilized on account of their reliability, validity, and current use in nutritional epidemiology. Some additions were made to the serving sizes, based on existing foods on the market to quantify the ultra-processed foods.

### 2.2. HISS Pilot Testing

Pilot testing was undertaken by the research team which included seven personnel all involved in nutrition-related research, including two senior nutrition researchers and practitioners. The team recorded and analyzed a variety of personal food records using the first draft of the HISS. Following this evaluation, the team made multiple adjustments and additions to the food-classification system, ensuring it comprehensively covered common food products.

### 2.3. Reliability Study: User Testing of the HISS Classification System

#### 2.3.1. Study Design

Five hypothetical 24 h food recalls were designed and captured using digital photography (Supplementary Materials). Each recall included meals from breakfast, lunch, and dinner, along with snacks and beverages consumed throughout the day. Recalls showcased a range of dietary patterns from predominantly whole foods to more processed items. Each meal/food item was photographed alongside a standard bank/debit/credit card for size reference to aid with easy identification of serving sizes. Each food record was collated into a single document. The document included every meal, snack, and beverage, each with its serving size noted to aid in accurate data collection. An online instructional video was created to demonstrate the use of the HISS classification table and use of serving sizes. The video also explained the overall aim for the research and food-classification activity. Participants were additionally provided with a reference list of the national food guidelines’ serving sizes to help guide them with determining food and beverage quantities in their food records.

Thirteen participants were included in a food-classification activity to assess the reliability of the HISS. Participants were all working nutrition health professionals in New Zealand who were familiar with dietary records and had a robust knowledge of nutrition and food composition. The sample size was selected based on power calculations of the minimum sample required to run the ICC analysis after consultation with a statistical expert. Ethical approval was obtained from AUTEC in September 2021 (approval number: 21/326). All participants were sent an information sheet, and consent form, and they provided written consent prior to the commencement of the research.

#### 2.3.2. Reliability Assessment Protocol

As this study was carried out remotely due to COVID restrictions at the time, participants were sent the HISS instructional video, HISS table, and five food recalls via email. After watching the instructional video, participants were asked to analyze each of the five food recalls using the HISS. The scoring sheet (Supplementary Materials) was completed by each participant with each serving of food and drink tallied and classified into the four HISS categories: unprocessed and minimally processed foods [category 1], processed I [category 2], processed II [category 3], and ultra-processed [category 4]. For each food and drink item, participants identified the extent of food processing according to the HISS category definitions and used the serving size examples listed for each food group to quantify the amount. The total servings for each food recall were also recorded on the scoring sheet.

Following the classification activity, participants were asked to complete questionnaires composed of three open-ended questions which were designed to gain insight into the usability of the HISS:

(1) What did you find easy to use?
(2) What did you find difficult to use?
(3) What are your suggestions for improvements?

The primary researcher additionally analyzed each of the five food records using the HISS and related documents; this was then verified by the nutrition research team. These scores were used as reference points for comparing the participants’ assessments.

### 2.4. Data Analysis

Descriptive statistics were used to examine the participants’ serving scores in each HISS category for the five food records. The mean and standard deviation of the food and drink servings were computed for each HISS category and food record, with the total servings also presented. To assess whether nutrition professionals could accurately categorize food from the digital food records, the percentage of overall servings in each HISS category was computed and compared to the research team’s assessment of the same five diets. Differences between the participant scores and the research team’s scores were calculated across each HISS category and for the total servings. Intraclass correlation (ICC) using a two-way mixed-effect model with a single rater type was then calculated to determine the inter-rater reliability between all scores collected during the activity. All ICC and descriptive statistics were computed in SPSS statistics software version 28.

To investigate the relationship between nutrient composition and the HISS categories, the nutrient values of each of the five food records were assessed using FoodWorks10 Professional (v10.0., Xyris Pty Ltd., Brisbane, Australia, 2019). The purpose of developing this system was to move away from nutrient-based analysis. However, investigating this aspect was important to assess the system’s plausibility. For example, if HISS 4 foods were found to be the highest in fiber and low in sugar then our system would likely have required further refinement. In each case, total energy (Kcal), total sugars (grams), total dietary fiber (grams), and total carbohydrates (grams) were calculated. These values were divided by the total number of servings estimated for each food record (averaged across all participants) to account for the differences in food quantity between the five records. The association between nutrient values and the percentage of servings in the unprocessed (HISS 1) and ultra-processed (HISS 4) categories was analyzed. This was completed for estimates made by each of the 13 participants, using repeated measures of Pearson’s correlation coefficients (r) with the rmcorr package in R (version 3.6.1 (7 May 2019)). We also included 50 foods from each of the HISS 1 and HISS 4 categories in this analysis. This ensured that no particular food category was omitted from the five-diet-plan analysis, thereby allowing for a broad range of foods. These foods were selected at random from the 2018 New Zealand Food Composition Database using a random number generator. Prior to selection, all duplicate items within the list of food items were removed. Mann–Whitney rank–sum tests were employed as non-parametric alternatives to a *t*-test due to non-normal data. These tests examined differences in each nutrient between the HISS 1 and HISS 4 groups using SigmaPlot version 14.5.

Finally, an inductive thematic analysis was conducted on participants’ feedback from the questionnaire, which contained open-ended questions. This analysis was used to explore themes identified within the qualitative data.

## 3. Results

Thirteen participants (eleven females, two males) completed the HISS classification activity (Table 2). Most were able to correctly identify which of the four HISS categories food belonged to, showing only slight differences from the primary researcher’s assessment. Participant 8 encountered challenges, however, notably in the unprocessed category (18-point difference) and processed II category (−14-point difference). While differences between participants and the researcher were relatively consistent across HISS categories, a few had larger disparities in the processed II category. Overall, participants exhibited consensus in classifying food into the four HISS groups. ICC analysis found a high degree of inter-rater reliability for both the unprocessed (0.778 (0.522–0.968)) and ultra-processed (0.82 (0.601–0.976)) categories. In contrast, the reliability of the two middle categories was lower (processed I = 0.226 (0.42–0.751), processed II = 0.066 (−0.30–0.537).

**Figure 1:**
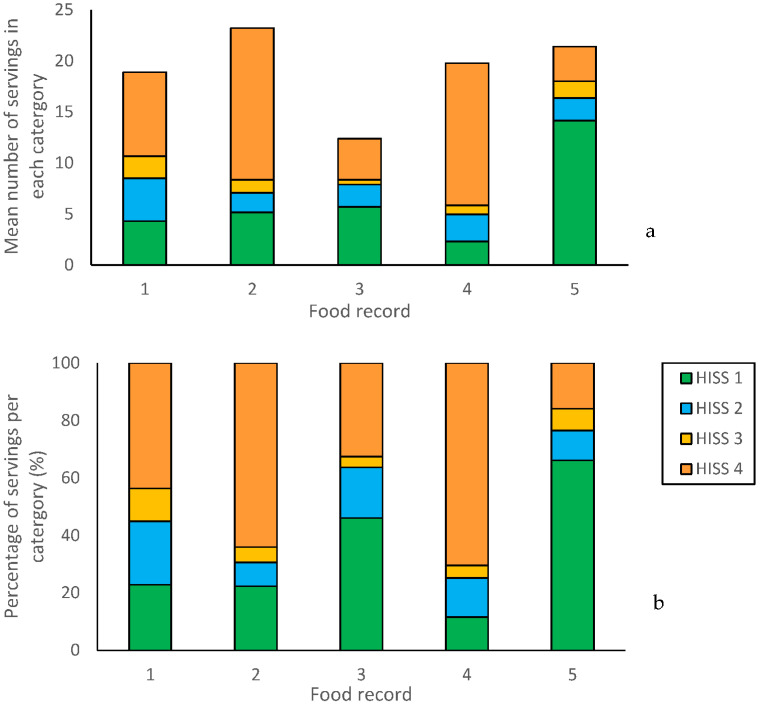
(**a**) Mean number of servings participants classified into each HISS category. (**b**) Mean percentage of total servings that participants classified to each HISS category. In both cases, HISS 1 = unprocessed, HISS 2 = processed I, HISS 3 = processed II, and HISS 4 = ultra-processed.

Participants rated the food record 5 as the least processed, with 14.17 (±3.68) servings identified as belonging to the unprocessed category (66% of total servings), while only 3.42 (±1.91) servings were assigned to the ultra-processed category (10% of total) (Figure 1). In contrast, food records 2 and 4 had high proportions of servings assigned to the ultra-processed category (64% and 70% of total servings, respectively). Food records one and three were classified as diets falling between these two extremes.

Table 3 presents nutrient composition (total energy, total sugar, dietary fiber, and total carbohydrates) for each food record. Overall, broad nutritional patterns were captured via HISS category classification. Notably, food record 5, the least processed, had the highest dietary fiber (50 g), while food records 2 and 4, with the highest percentage of ultra-processed servings, had the highest total sugar (330 g and 331 g, respectively).

### 3.1. Relationship between HISS Categories and Nutritient Composition

With reference to the five food records, consuming a higher proportion of foods in the ultra-processed HISS 4 category was positively correlated with greater total energy, rrm (51) = 0.76, *p* < 0.001, total carbohydrates, rrm (51) = 0.69, *p* < 0.001, and total sugar, rrm (51) = 0.78, *p* < 0.001. In contrast, there was a negative correlation between a higher proportion of foods in the HISS 4 category and dietary fibre, rrm (51) = −0.63, *p* < 0.001. The reverse was true for the unprocessed HISS 1 category, with energy (rrm (51) = −0.83, *p* < 0.001), carbohydrates (rrm (51) = −0.84, *p* < 0.001), and sugar (rrm (51) = −0.62, *p* < 0.001) showing negative associations, while dietary fibre was positively correlated (rrm (51) = 0.46, *p* < 0.001).

**Figure 2:**
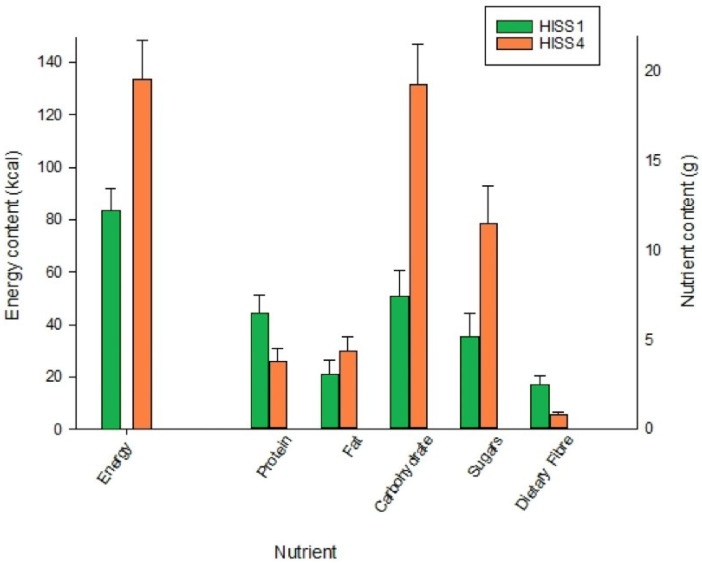
Mean nutrient content for 50 foods selected at random from HISS 1 and HISS 4 groups. Energy (kcal) corresponds to the left-hand *y*-axis, while all other nutrients (grams) correspond to the right-hand *y*-axis. Error bars denote the standard error of the mean.

Analysis of a wide range of food items showed similar results, with significantly higher energy content (U = 866.00, *p* = 0.008), carbohydrates (U = 601.50, *p* < 0.001), and sugar (U = 841.50, *p* = 0.005) in HISS 4 food items, than in HISS 1 food items. Dietary fiber was, in contrast, significantly higher in HISS 1 foods (U = 781.00, *p* < 0.001), while there was no significant difference in fat (U = 1000.50, *p* = 0.086), or protein content (U = 1030.50, *p* = 0.131), between HISS 1 and 4 foods. Mean values for the two HISS groups are displayed in Figure 2.

### 3.2. Questionnaire Results

Table 4 presents the themes and supporting transcripts from responses derived from the open-ended questions. In general, participants found the system easy to use. Where confusion or suggestions for improvements occurred, it was most often related to particular hard-to-place mixed-food items, which could be placed in multiple categories dependent on their exact ingredients.

## 4. Discussion

The study demonstrated that the HISS, a modified NOVA food-classification system using digital photography for food recalls, is both reliable and practical. This system identifies diet quality based on the level of food processing. Our findings indicate that trained nutritional professionals were able to effectively assess food records using digital photography images. The method clearly differentiates between high- and low-quality foods and diets. This suggests that the HISS could be a valuable tool. It assesses both the quantity and quality of diets without relying on nutrient-based analysis. We observed high inter-rater reliability in the two critical outer HISS categories: unprocessed (category 1) and ultra-processed foods (category 4), with a general agreement among most participants in classifying foods. Moreover, the HISS was found to capture meaningful relationships between the level of food processing and nutrient composition. While the purpose of this system is to move away from a nutrient-based analysis, we demonstrate its plausibility. This is shown through its ability to predict traditional elements of nutrient-based diet quality. Additionally, the study also evaluated the usability of the HISS through quantitative measures. To our knowledge, this is the first food-classification system that has explicitly aimed to assess a measure of food quality in a quantitative manner, utilizing digital photography.

While several other food-classification systems based on the level of food processing already exist, and, in the case of NOVA, have been widely utilized [24,26], all of these current classification systems have inherent limitations [26]. Quantifying the number of servings in each food processing classification group presents a significant challenge. This is compounded by the difficulty in classifying home-cooked items and more complex dishes with multiple ingredients Additionally, self-reporting of diet has frequently been found to be inaccurate and is associated with significant bias depending on the underlying health of individuals [4]. By utilizing digital imaging and including details of both serving size and home-cooked dishes within the HISS framework, we overcome these limitations. This approach represents a significant strength of our method. Accordingly, the framework allows for more accurate assessment of overall patterns in diet by both individuals and nutrition professionals.

Reliability of the framework was a primary objective of the current research. The observed high degree of inter-rater reliability among participants was encouraging, particularly with regard to the unprocessed and ultra-processed HISS categories. Secondly, being able to identify both whole foods that are essential for good health, and UPFs that are associated with poor health, was achieved. These categories represent the clearest representation of high- and low-quality diets when the food matrix is considered. While there was lower inter-rater reliability of the processed I (HISS category 2) and processed II (HISS category 3) groups, participant feedback was utilized to modify and improve the HISS, hopefully leading to a clearer distinction between these two categories in future. We contrast these results with previous validation studies of the NOVA classification, which only found a moderate agreement between raters [36], even when ingredient lists were given [7]. Accordingly, while we acknowledge that refinements are required to improve the distinction between HISS 2 and HISS 3, this should not be a barrier to using a modified version of the HISS moving forwards.

While the purpose of this study was not to undertake a comparison between the HISS and the NOVA systems, we did classify the five food recalls according to NOVA out of interest. When looking across the combined results of all five records, we found good agreement between group 1 and 4 (as we expected), but found very different results for groups 2 and 3 (again, as expected, especially as NOVA group 2 comprises culinary ingredients exclusively). Further work to assess the alignment of these two systems using a wider dataset of meal plans is warranted.

The results from our exploration of nutrient analysis also support the notion that the HISS can be used as a classification system to quantify the quality of foods. It is able to act as a reasonable proxy for less-controversial standard nutrient measures including fiber and sugar content. We found consistent associations between the HISS score and traditional nutrient measures including sugar, fiber, and total energy content. Ultra-processed foods were associated with higher energy content, higher total sugar, and higher total carbohydrates, as compared to non-processed and minimally processed foods. In contrast, a high fiber level was predictive of the unprocessed category. These results concur with the previous research linking UPFs with high sugar and carbohydrate content, but low fiber [22,38]. While more traditional methods of diet analysis allow nutrients to be quantified, HISS avoids many of the errors implicit in these more reductionist methods. Specifically, traditional methods that combine all macronutrients into a single category lose information about the structure of the food matrix. For example, they miss details like the quality of carbohydrates, their glycemic index, and the overall metabolic effects of diet [35].

Participant feedback was extremely helpful in identifying areas of HISS which required adjustment and informing development of the system moving forward. Issues surrounding serving sizes, food item classification, and HISS table layout and formatting underscored the need for clearer guidelines. This was particularly evident for foods with intricate serving sizes. The subjective nature of categorization is somewhat unavoidable, as exemplified by artisanal and homemade bread. This suggests a need to refine classification criteria, especially for the processed I and II groups. Aligning the system’s purpose with health outcomes emerged as a vital consideration and something that will help to guide further modification of the framework. These insights offer valuable direction for potential enhancements in serving size guidelines and classification criteria; the goal being to create a more effective tool for evaluating food processing levels and their impact on health.

The development of an accurate and reliable tool for measuring the quality of overall dietary patterns has significant public health implications. As noted, unhealthy diets posit a significant and ever-increasing public health issue. In 2017, 11 million deaths globally were attributed to unhealthy diets, with malnutrition in all forms costing up to 3.5 trillion a year [1]. Helping individuals to improve their nutrition literacy and make better informed food choices is, therefore, imperative, and a system such as HISS could be instrumental in this. HISS has the potential to aid individuals in becoming more aware of their dietary habits, drawing attention to the need to consume fewer ultra-processed foods, and guiding them towards quality whole foods and home-cooked meals. The simplicity and useability of the system is a key strength in this regard, particularly given the use of digital photography rather than complicated food diaries and recalls. A HISS app employing AI technology is currently under development by this research group. This will make the system easily accessible to anyone with a smartphone, thereby extending health promotion initiatives to a broader community. Further, the system can be used by nutrition professionals working with Food Frequency Questionnaires (FFQs) in nutritional epidemiology research. By integrating HISS, FFQs may more effectively analyze the impact of processed foods on health and identify dietary patterns, especially concerning processed-food consumption.

The HISS framework also has the potential to be used to provide further education around the abundance of UPFs in society and promote policy change around dietary recommendations. A shift away from diets centered around UPFs further has the potential to create positive environmental change. While the current food systems are responsible for 20–35% of greenhouse gas emissions and approximately one-third of global land use; environmental pressures are predicted to increase further as the population rises to 9.7 billion by 2025 [2,39]. A food production system dominated by UPFs has a severe impact on eco-planetary health. These foods require more natural resources and have a larger environmental footprint compared to less-processed diets [40,41]. Intensive industrial agricultural activities and single-use plastic packaging additionally contribute to climate change and the global-plastic-waste crisis [42,43]. Promoting healthy and sustainable diets is, therefore, crucial for safeguarding both human well-being and the long-term health of the planet, as recognized by various international public health agencies and guidelines [1,2].

While HISS has several advantages over other systems currently in use, both the classification system and the study have several limitations. Firstly, due to the small-scale nature of this study, further research is required to validate and refine the modified version of HISS that was developed based on participant feedback from the current study. As only a limited number of food records and participants were involved in this pilot study, further testing with a greater number of food records and participants is desirable. Additionally, all the raters in the present study were nutrition professionals; we believe this was most appropriate as the first step in testing a new system. For the classification to be widely used, however, it is important that members of the general public are able to use the system. Allowing more diverse groups of participants to test the system using their own diets is, therefore, the next step in developing the HISS.

More fundamentally, given the complexities involved, all food-classification systems have inherent limitations. They must, by necessity, simplify a complex problem in order to be useable. Attempts to categorize food by its level of processing have been severely criticized by some [26,32,33]. We acknowledge that the system is not perfect. As pointed out by Tobias [44] and others, the ultra-processed group contains a diverse range of foods. Some of these foods are almost universally accepted as being detrimental to health, while others may be beneficial in specific contexts. As an example, high-quality protein powders and other sports nutrition products would be classed as ultra-processed. However, they often contain minimal ingredients and may be beneficial, or even essential to certain demographics, as could foods developed to address specific medical concerns. Foods fortified with functional ingredients represent another area of complexity. As Forde [45] points out, it is entirely unrealistic and perhaps not even desirable for the population to stop consuming all ultra-processed foods. This is because a significant amount of nutrients are currently derived from these foods in many countries [37]. Discussion around what constitutes a meaningful difference in the level of processing and how this relates to health is certainly needed [26]. We also note Hess’s [46] criticism that it is theoretically possible to build a ‘healthy’ diet (as defined by the 2020 Dietary Guidelines for Americans (DGA)) composed of >80% UPF. However, it is also clear that at a broad scale, the evidence that high UPF consumption is associated with poor health and diet quality is robust [12,13,14,15,16,17] and that a diet centered primarily on whole foods has numerous health benefits [1,19]. Therefore, promoting a diet with a reduced proportion of UPFs that includes more whole, unprocessed, or minimally processed foods is a desirable goal for improving population health. Further, we note that many of the foods classified as UPF by Hess et al. [46] (particularly canned fruits and vegetables) would not be defined as so by the HISS. Given the high levels of both public confusion and academic debate around specific nutrients, a system which presents clear information to the public is highly desirable. This is especially relevant in New Zealand, where 83% of food items available in supermarkets are UPFs [47]. Moreover, research suggests that the term ‘ultra-processed‘ is an increasingly well-understood concept and that most people are able to identify examples of UPFs [48].

## 5. Conclusions

In summary, this study offers an initial authentication of the Human Interference Scoring System (HISS). This is a novel approach that builds upon the NOVA system. HISS integrates digital photography with food recalls and uses food servings for quantification instead of individual nutrients. This approach could make HISS more user-friendly and less controversial compared to other diet-quality tools. HISS addresses certain limitations of current food-classification systems. However, its full effectiveness in promoting healthier diets and impacting public health requires further investigation and validation. Continued development and research are essential, including exploring a digital HSS app. These efforts are needed to fully utilize and understand this system’s capabilities.

## Figures and Tables

**Table 1:** Human Interference Scoring System (HISS). Food-classification system with group names and numbers indicated in bold text.

Food Groups and Definition	Examples
**1: Unprocessed and minimally processed** Raw and whole foods with little or no processing. Foods that are fresh, chilled, canned, frozen, or dried to enhance nutrients and freshness at their peak. Unprocessed foods are of plant and animal origin. Minimally processed foods are natural foods that are altered with removal of inedible or unwanted parts and preserved for storage. Foods that have not been through processing methods and are without added ingredients.	Fruit and vegetables; eggs; canned fish, meat, and legumes (beans, chickpeas, lentils) in spring water; red and white meats (beef, chicken, lamb, pork, venison); nuts and seeds; honey; herbal teas; water; soda water; herbs and spices.
**2: Processed I** Artisanal products that were typically available for consumption in pre-industrial societies. Products that require traditional processing techniques with bacteria fermentation (cultured products), yeast strains, and natural ingredients. Foods that have been processed for preservation and safe consumption but remain as single foods.	Milk; butter; cheese; milk and coconut creams; unflavoured yoghurts; sourdough and artisan bread; coffee beans; pasta; plain oats; shredded wheat; grains such as rice and corn; couscous and polenta; fermented alcoholic beverages (beer, cider, and wine); spirits; kombucha; broths; sauerkraut and pickled vegetables.
**3: Processed II** **3.1** Domestically assembled items, often prepared with separate ingredients including raw or whole food products with additional cooking agents to produce meals, dishes, or snacks. **3.2** Foods processed for preservation with additional flavouring and additives with no further cooking needed. Whole foods with more extensive methods of preservation such as salting, salt-pickling, smoking, and curing. Includes canning and bottling techniques using sugar or syrups, oil, or additional flavouring.	**3.1** Granola and breakfast cereals; baking and biscuits; homemade plant-based milk; soup. **3.2** Fruit preserved in syrup; canned vegetables and legumes preserved in brine; canned fish in flavouring or oil; processed and cured animal foods (ham, bacon, pastrami, beef jerky, bacon); salted or sugared nuts and seeds; hummus; pesto; aioli; nut butters; pasta sauces; fortified wine; plant-based milks.
**4: Ultra-processed** Industrially prepared items that are largely manufactured and packaged which are ready to eat at home or at fast food outlets. Undergone high degrees of processing entirely from substances that are derived from foods, with little or no whole foods present. Formulations of ingredients with industrial techniques and processes. Contain additives to prolong product duration, including varieties of sugars (corn syrup, maltodextrin, dextrose, lactose), modified oils, and sources of protein (hydrolysed proteins, soya protein isolate, gluten, casein, whey protein). Contain large amounts of additives, preservatives, stabilisers, emulsifiers, bulkers, artificial sweeteners, thickeners, colors, and flavours.	**4.1** Ready-to-consume products: mass-produced packaged breads, buns, and wraps; biscuits; baked beans or spaghetti; cakes; confectionery (chocolate, candy); instant coffee sachets; milo and hot chocolate; rice cakes; ice cream; breakfast cereals; packaged snack products (e.g., chips); fizzy and energy drinks; sweetened milk drinks; sweetened fruit yoghurt; juice; powdered and packaged soups and desserts; noodles; muesli bars; protein supplements and bars; instant dressings and sauces; spreads; margarine; baby formulas; ready-to-drink alcoholic beverages; tonic water. Pre-prepared ready-to-heat products: poultry and fish ‘nuggets’ and ‘sticks;’ sausages, burgers, hot dogs, and other reconstituted meats; packaged foods (pizza, burgers etc); fries; pies.

**Table 2:** Percentage of servings in each HISS category as estimated by the primary researcher (red) and 13 health professionals. Differences show the percentage-point difference between the estimates of the primary researcher and those of each participant. Bold values indicate differences of more than 10 percentage points. Values in parentheses indicate standard deviations of the mean.

	% Overall Servings in Unprocessed Category	Difference	% Overall Servings in Processed I Category	Difference	% Overall Servings in Processed II Category	Difference	% Overall Servings in Ultra-Processed Category	Difference	Total Servings	Difference
**Research team**	**36**		**16**		**1**		**47**		**94.5**	
Participant 1	36	0	13	3	0	1	51	−4	83.0	**11.5**
Participant 2	37	−1	20	−4	6	−5	38	9	90.2	4.3
Participant 3	33	3	21	−5	0	1	46	1	87.0	7.5
Participant 4	34	2	14	2	5	−4	47	0	100.5	−6.0
Participant 5	39	−3	8	8	2	−1	51	−4	106.0	**−11.5**
Participant 6	32	3	9	7	10	−9	49	−2	89.3	5.3
Participant 7	34	2	10	5	5	−4	50	−3	106.0	**−11.5**
Participant 8	18	**18**	20	−4	15	**−14**	48	−1	88.0	6.5
Participant 9	31	5	12	4	4	−3	53	−6	101.7	−7.2
Participant 10	33	3	10	6	9	−8	47	0	99.0	−4.5
Participant 11	30	6	14	2	18	**−17**	38	9	103.0	−8.5
Participant 12	33	3	11	4	5	−4	51	−3	92.0	2.5
Participant 13	37	−1	20	−4	6	−5	37	**10**	99.8	−5.3
Mean	33.8 (SD 5.1)		13		7		47		95.8 (SD 7.8)	

**Table 3:** Nutrient composition of food records.

Food Records	Total Energy (Kcal)	Total Sugar (g)	Dietary Fiber (g)	Carbohydrate (g)
1	2602	90	41	346
2	3271	230	6	330
3	1179	43	10	109
4	2617	166	18	331
5	2002	97	50	162

**Table 4:** Themes and quotations from qualitative analysis.

Theme/Sub-Theme	Quotes from Transcript
HISS categories	My main issue was categorising the food items in their correct classification, as I got a little confused. For example, you have “artisan breads” on Processed 1 but also “homemade bread” on Processed 2. For me, these two items would be categorised the same. If the desired outcome is a tool for ‘quality’ some of the categories may be incongruous. For example, brought pasta sauces should (according to my reading of your list) be ultra-processed but if these include minimal added ingredients (esp. sugar) they would be considered a highly nutrient-dense food and one associated with improved outcomes. For example, spread (such as peanut butter and jams, or even salad dressing and hummus) could easily fall in different categories, depending on how they are made. For example, shop-brought jams usually contain only fruit and sugar, plus some preservatives as pectin, which according to NOVA is not an ultra-processed type of additive. Some nut butters are 100% nuts and maybe salt, whereas more processed ones will contain emulsifiers, which is a type of additive that only appears in ultra-processed foods according to NOVA. I did not find it easy to use, and to me it seems a bit arbitrary. “Like beer processed 1, but health wise I’d put it in ultra-processed”.
Potential areas of improvement	You could note what items what people put where/serving size per items to check exactly how people are using the system. On the Classification sheet, perhaps place the Serving Size column in a separate box, or underneath the classification, to make it easier to read the document. Right at the start, when looking for an item’s classification, I spotted the same item on the serving size column and automatically looked for the category in that row, but then realised they were two sections apart, (1). Classification + Examples; and (2). Serving Sizes.
Positive comments	It uses a nutrient-density focus rather than energy intake approach which resonates well with me. Useful to see it laid out in the end and what meal components added up to for a days’ worth of food.The photographs give a really easy way to identify what’s eaten in a day and how processed the food item is. I found it easy to have the picture and text to verify what the food was. For example, I could see who clearly had half a chicken for lunch and could visually estimate how many servings of chicken that might be. With text only, chickens vary in size.

## Data Availability

Data are contained within the article and Supplementary Materials.

## Supplementary Materials

The following supporting information can be downloaded at: https://www.mdpi.com/article/10.3390/nu16040536/s1, Five hypothetical food recalls showing all food and liquid consumed over 24 h. Participant scoring sheet.
