# Supplementary material for: The Human Interference Scoring System (HISS): A New Tool for Quantifying Food Quality Based on Its Level of Processing
Source: Nutrients. 2024 Feb 14;16(4):536. doi: 10.3390/nu16040536 (PMC10892936; doi:10.3390/nu16040536)
Supplement: Supplementary file 1 [file nutrients-16-00536-s001.zip › HISS 5 Food Recalls.pdf]

Food Record 1

Meal 1

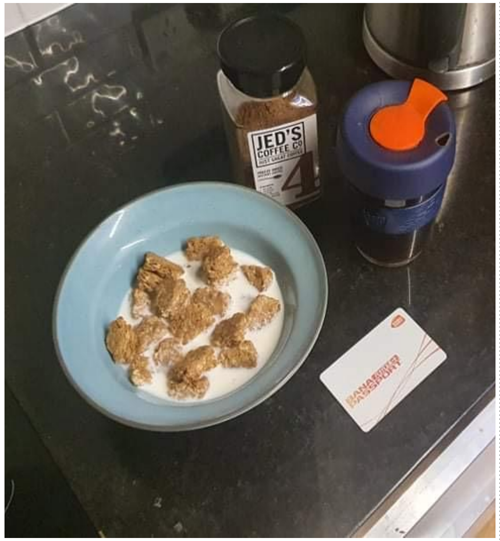

<u>Breakfast</u>
1 cup of blue-top milk
2 cups of Honey Weet-bix bites

<u>Beverages</u>
1 cup of instant black coffee, no milk or sugar added
1 lion red beer 330ml

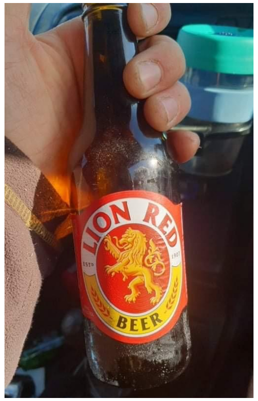

Meal 2

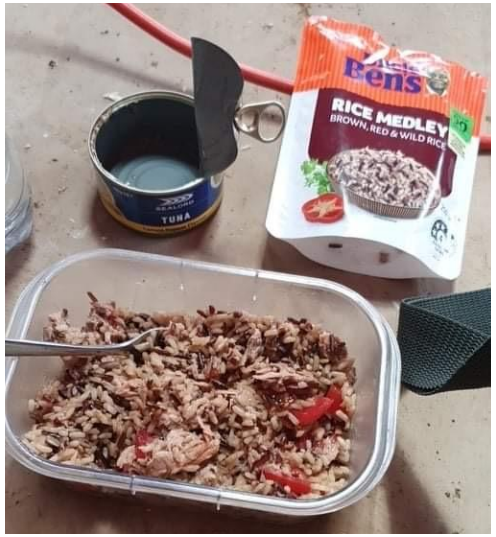

<u>Lunch</u>
250g/ 1 packet of uncle bens rice medley
95g lemon pepper canned tuna

<u>Snacks</u>
1 medium banana
1 medium mandarin
1 protein nut bar
1 olive oiled seaweed packet

Meal 3

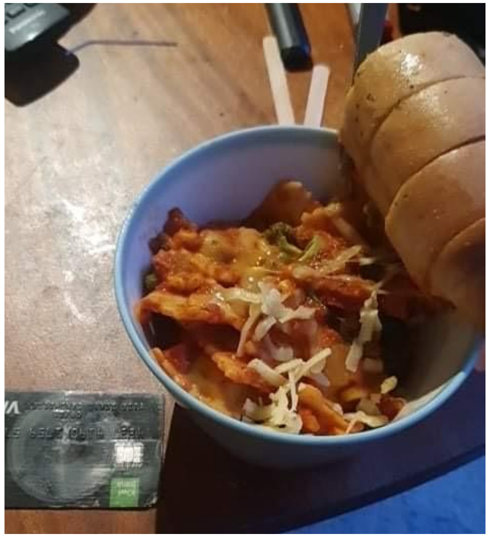

<u>Dinner</u>
1 and a ½ cups of cooked tomato basil and mozzarella ravioli
½ cup dolmio garlic pasta sauce
1 tsp of colby cheese
28g of chorizo
1 cup of cooked vegetables (peas, corn, spinach, mushrooms, capsicum)
⅓ store brought garlic bread

Snack / Other

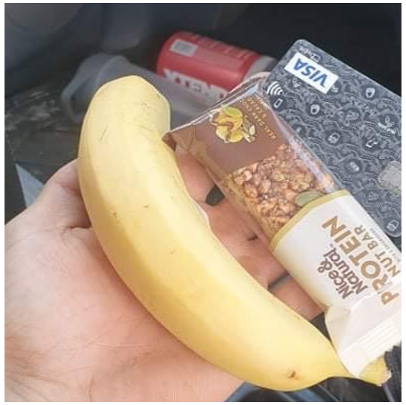

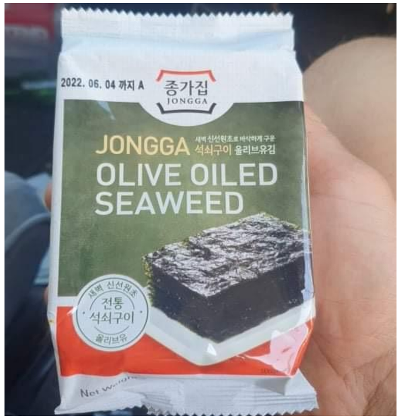

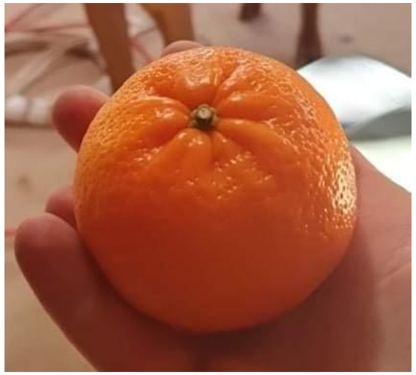

Food Recall 2

Meal 1	Meal 2	Meal 3	Snack / Other
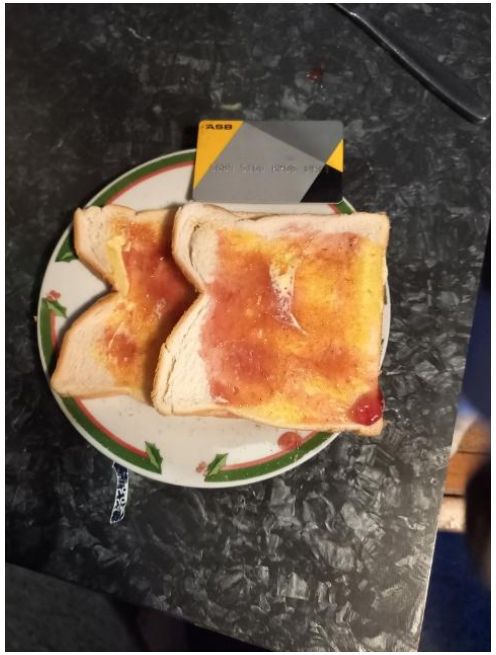	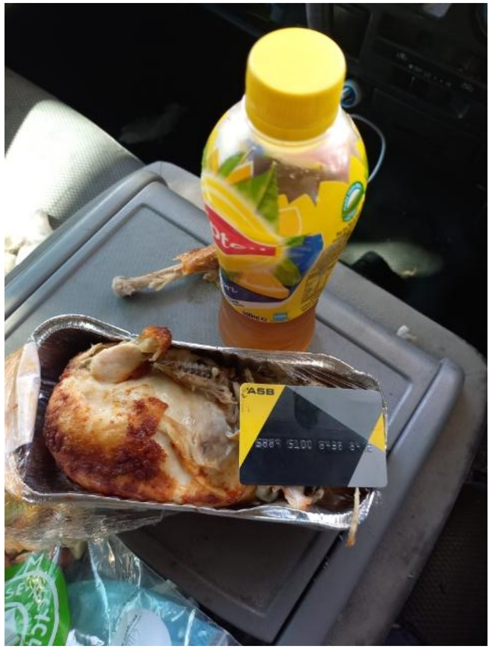	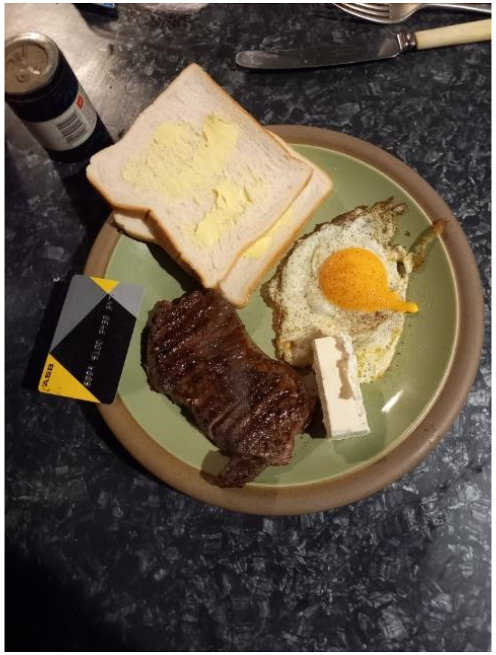	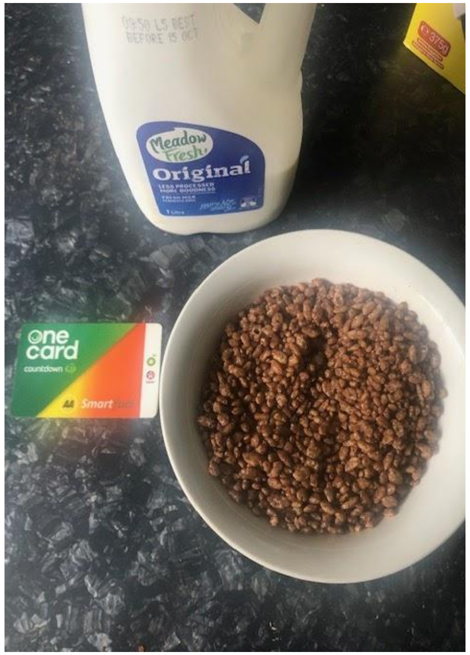<br>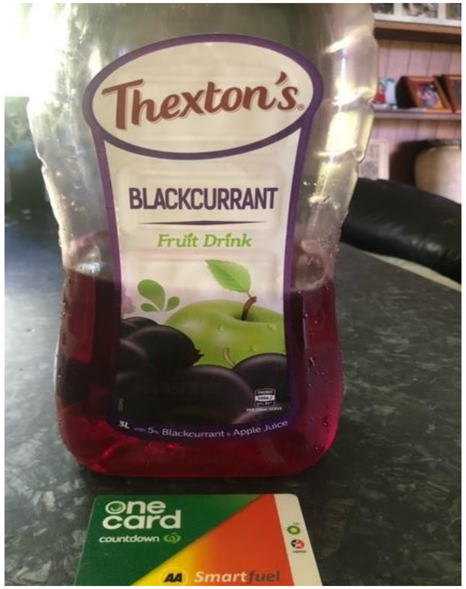
<u>Breakfast</u><br>2 slices of white bread<br>2 tsp olivani margarine<br>2 tsp of raspberry jam	<u>Lunch</u><br>Half an oven-baked chicken<br><br><u>Beverages</u><br>500ml Lipton iced tea<br>1.5litres of blackcurrant fruit drink	<u>Dinner</u><br>2 slices of white bread<br>2 tsp of olivani margarine<br>1 fried egg<br>200g fried steak<br>1 slice of Feta<br><br><u>Snacks</u><br>2 cups of coco pops<br>1 cup of blue-top milk	

<table>
<tr><th colspan="4">Food recall 3</th></tr>
<tr><th>Meal 1</th><th>Meal 2</th><th>Meal 3</th><th>Snack / Other</th></tr>
<tr>
<td>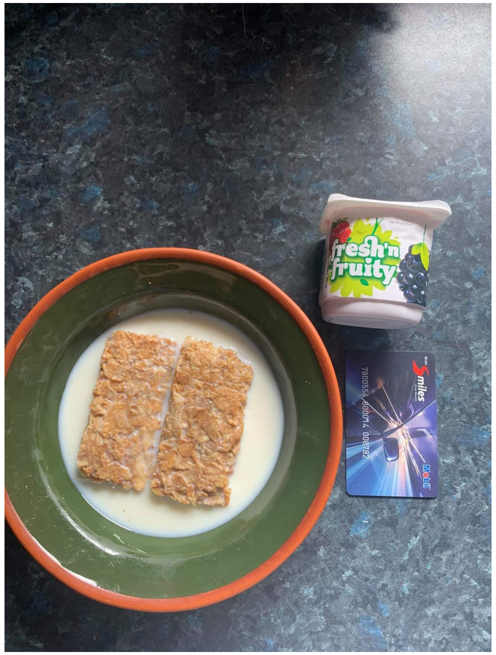

<u>Breakfast</u>
2 weet-bix
1 cup of blue-top milk
1 small tub of sweetened fruit yoghurt</td>
<td>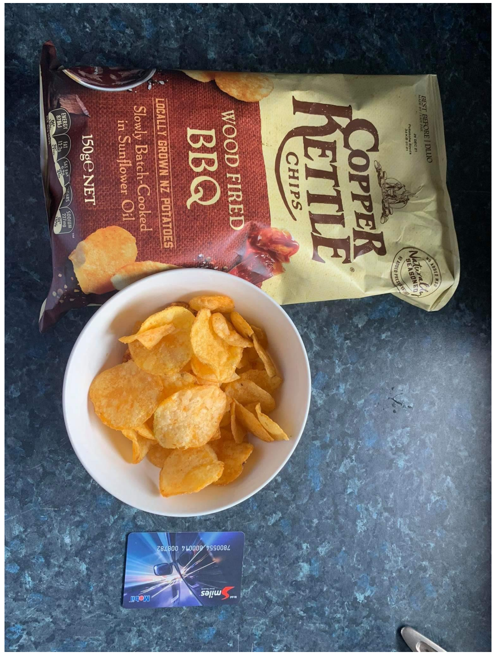

<u>Lunch</u>
⅓ bag of copper kettle bbq flavoured chips</td>
<td>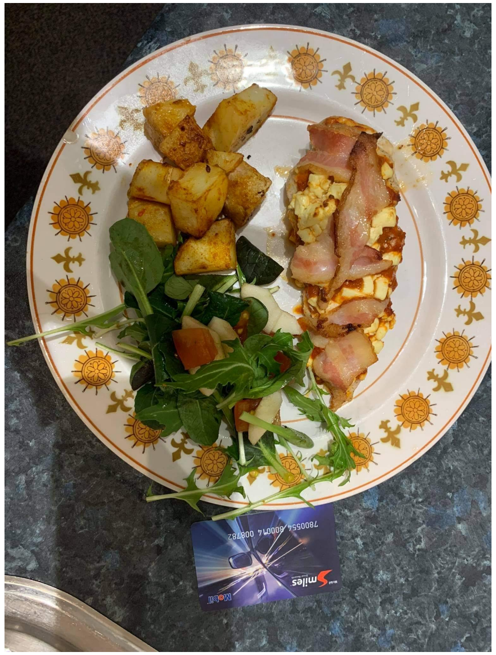
<u>Dinner</u>
200g baked chicken breast
1 slice of bacon
2 slices of feta
1 baked potato
½ cup of salad leaves
¼ cup of pear and tomato</td>
<td></td>
</tr>
</table>

Food Recall 4			
**Meal 1**	**Meal 2**	**Meal 3**	**Snack / Other**
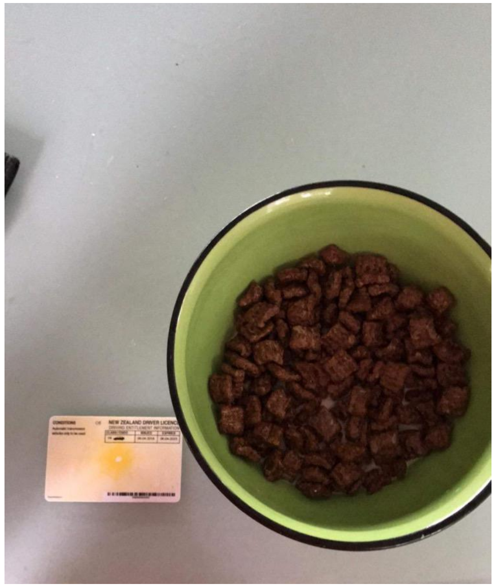<br>Breakfast<br>3 cups of milo cereal<br>2 cups of blue-top milk	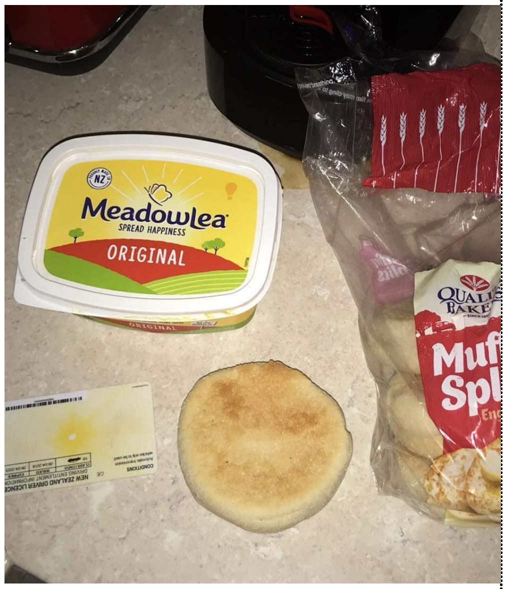<br>Lunch<br>4 muffin splits<br>2 tsp of margarine on each muffin	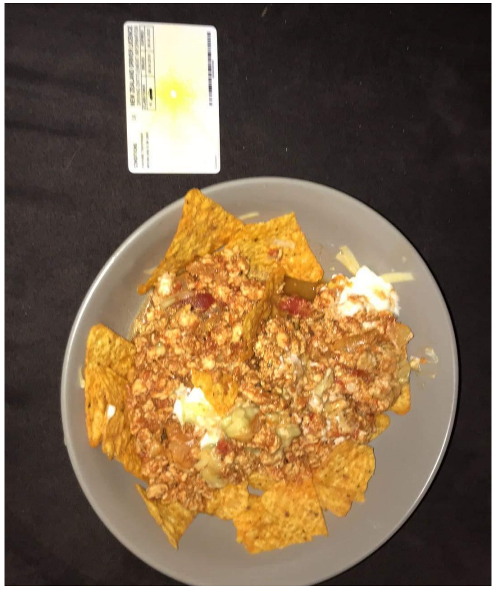<br>Dinner<br>150g chicken mince<br>⅓ canned tomatoes<br>2 tbsp sour cream<br>2 handfuls of doritos salsa corn chips	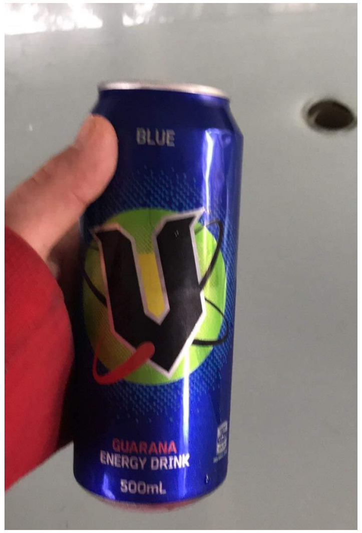<br>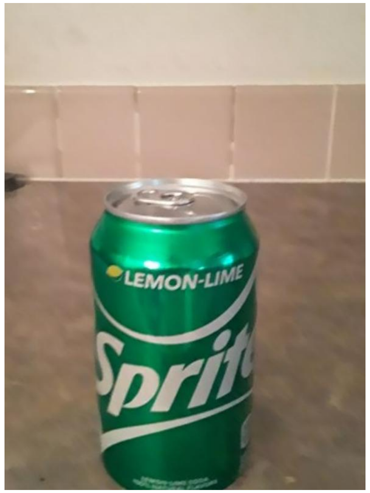<br>Beverages<br>500ml Large blue V<br>330ml Sprite

Food recall 5			
**Meal 1**	**Meal 2**	**Meal 3**	**Snack / Other**
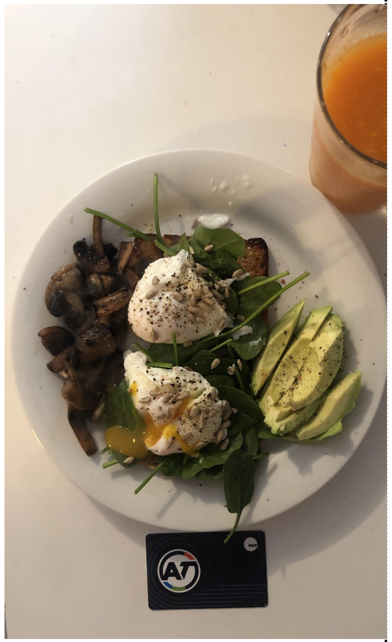<br>Breakfast<br>2 thin vogels mixed grain toast<br>½ avocado<br>½ cup spinach<br>2 poached eggs<br>½ cup fried mushrooms<br>2 tsp sunflower seeds<br><br>Beverages<br>500ml freshly squeezed orange, carrot, and apple juice	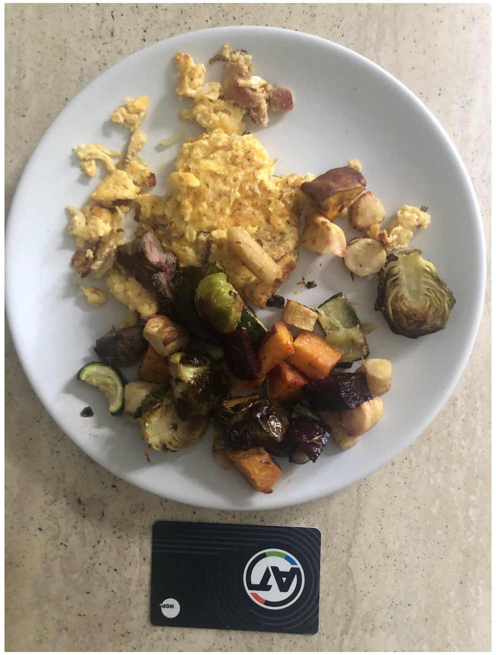<br>Lunch<br>1 scrambled egg with colby cheese<br>2 handfuls of roasted vegetables<br><br>Beverages<br>Medium takeaway coconut milk cappuccino	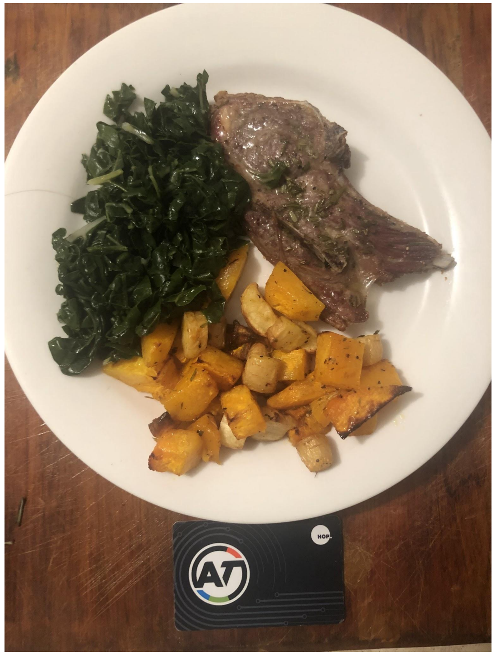<br>Dinner<br>1 oven-baked lamb chop<br>1 cup of boiled silverbeet<br>1 cup of roasted kumara parsnip and butternut	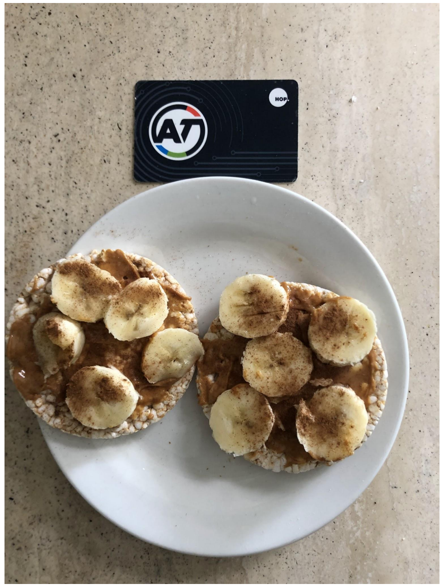<br>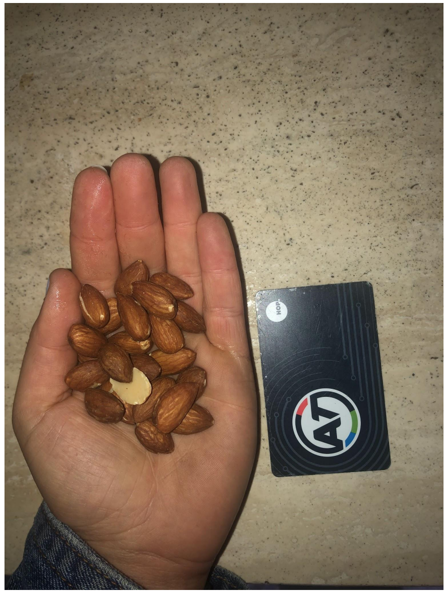

	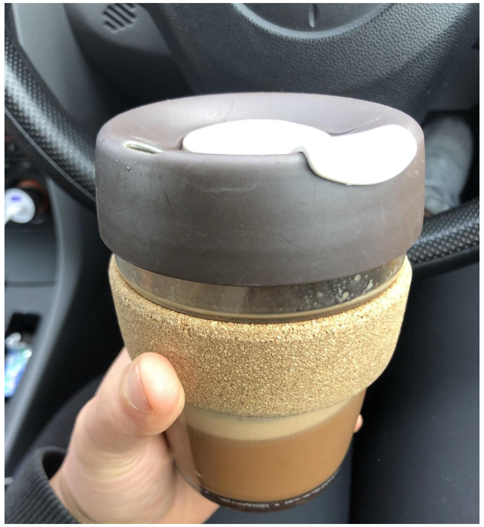		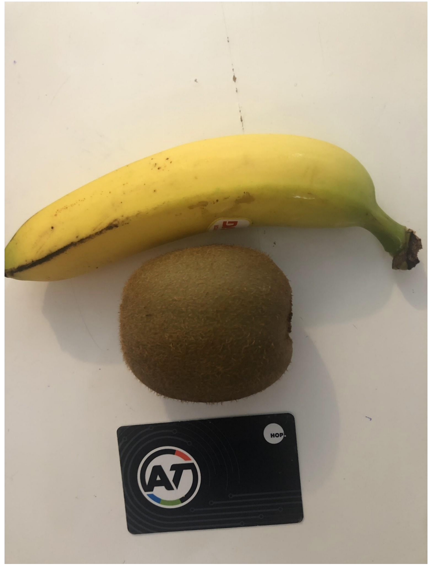<br><u>Snacks</u><br>2 brown rice cakes with quinoa<br>3 tsp of fix and fogs peanut butter<br>1.5 medium banana<br>1 handful of roasted unsalted almonds<br>1 small kiwifruit
