# Supplementary material for: The Human Interference Scoring System (HISS): A New Tool for Quantifying Food Quality Based on Its Level of Processing
Source: Nutrients. 2024 Feb 14;16(4):536. doi: 10.3390/nu16040536 (PMC10892936; doi:10.3390/nu16040536)
Supplement: Supplementary file 1 [file nutrients-16-00536-s001.zip › HISS Scoring Sheet.pdf]

TE WĀNANGA ARONUI O TĀMAKI MAKAU RAU

# Food Classification Activity Scoring Sheet

Date: _______________

Name: ___________________________________________________________________________

| | 1: Unprocessed/ Minimally processed | 2: Processed I | 3: Processed II | 4: Ultra-processed |
|---|---|---|---|---|
| No. 1 | | | | |
| **Tally of Servings** | | | | |
| **Total Servings** | | | | |
| % Total | | | | |
| No. 2 | | | | |
| **Tally of Servings** | | | | |
| **Total Servings** | | | | |
| % Total | | | | |
| No. 3 | | | | |
| **Tally of Servings** | | | | |
| **Total Servings** | | | | |
| % Total | | | | |
| No. 4 | | | | |
| **Tally of Servings** | | | | |
| **Total Servings** | | | | |
| % Total | | | | |
| No. 5 | | | | |
| **Tally of Servings** | | | | |
| **Total Servings** | | | | |
| % Total | | | | |

Please provide feedback on the usability of the HISS classification and scoring system with responding to the following questions:

What did you find easy to use?

……………………………………………………
……………………………………………………
……………………………………………………
……………………………………………………
……………………………………………………
……………………………………………………
……………………………………………………
……………………………………………………
……………………………………………………
……………………………………………………
……………………………………………………
……………………………………………………
……………………………………………………
……………………………………………………
……………………………………………………

What did you find difficult to use?

……………………………………………………
……………………………………………………
……………………………………………………
……………………………………………………
……………………………………………………
……………………………………………………
……………………………………………………
……………………………………………………
……………………………………………………
……………………………………………………
……………………………………………………
……………………………………………………
……………………………………………………
……………………………………………………
……………………………………………………

What are your suggestions for improvements?

……………………………………………………
……………………………………………………
……………………………………………………
……………………………………………………
……………………………………………………
……………………………………………………
……………………………………………………
……………………………………………………
……………………………………………………
……………………………………………………
……………………………………………………
……………………………………………………
……………………………………………………
……………………………………………………
……………………………………………………
……………………………………………………
